# Fused Deposition Modeling of Polymer-Based Magnetic Composites from Recycled Permanent Magnets of Discarded Hard Drives

**DOI:** 10.3390/ma19112356

**Published:** 2026-06-02

**Authors:** Duccio Gallichi-Nottiani, Daniel Milanese, Fausto Franchini, Emir Pošković, Marco Actis-Grande, Marta Ceroni, Luca Ferraris, Claudio Sangregorio, Claudia Innocenti, Martin Albino, Andrea Caneschi, Corrado Sciancalepore

**Affiliations:** 1 Department of Engineering and Architecture, University of Parma, Parco Area delle Scienze 181/A, 43121 Parma, Italy; duccio.gallichinottiani@unipr.it (D.G.-N.); daniel.milanese@unipr.it (D.M.); 2 National Interuniversity Consortium of Materials Science and Technology (INSTM), Via G. Giusti 9, 50121 Florence, Italy; fausto.franchini@polito.it (F.F.); emir.poskovic@polito.it (E.P.); marco.actis@polito.it (M.A.-G.); marta.ceroni@polito.it (M.C.); luca.ferraris@polito.it (L.F.); claudio.sangregorio@cnr.it (C.S.); claudia.innocenti@unifi.it (C.I.); martin.albino@iccom.cnr.it (M.A.); andrea.caneschi@unifi.it (A.C.); 3 Energy Department, Politecnico di Torino, Corso Duca degli Abruzzi 24, 10129 Torino, Italy; 4 Department of Applied Science and Technology, Politecnico di Torino, Corso Duca degli Abruzzi 24, 10129 Torino, Italy; 5 CNR-ICCOM, Via Madonna del Piano 10, 50019 Sesto Fiorentino, Italy; 6 Department of Chemistry—DICUS, University of Florence, Via della Lastruccia 3, 50019 Sesto Fiorentino, Italy; 7 Department of Industrial Engineering—DIEF, University of Florence, Via di S. Marta 3, 50139 Florence, Italy

**Keywords:** polymer-based magnetic composite, recycled permanent magnet, fused deposition modeling

## Abstract

Polymer-based composites with magnetic properties are promising materials that are able to combine the usual polymer features (low density, high electrical resistance, enhanced flexibility, and processability, etc.) with magnetic properties typically associated with ferro- or ferrimagnetic metals, alloys or metal oxide. The combination of recycled NdFeB powders with additive manufacturing techniques based on material extrusion enables the production of magnetic composites. The novelty of this approach lies in the use of 3D printing supported by an external magnetic field, which is used to align the particles during the printing process and thus improve the final magnetic properties. This approach represents a sustainable strategy for the recovery of electronic waste, converting it into high-value-added magnetic materials intended for additive manufacturing applications. Micrometric particles made of a Neodymium–Iron–Boron (NdFeB) alloy are compounded with a flexible thermoplastic matrix made of polybutylene adipate-co-terephthalate (PBAT). The NdFeB alloy is recovered from permanent magnets of obsolete hard drives and is demagnetized, ground to powder under an inert atmosphere, and finally sieved to a particle size below 50 µm. The obtained powder is mixed with the polymer using a twin-screw extruder. The composite material containing the NdFeB particles is then processed to obtain a calibrated filament, used for the fused deposition modeling (FDM) three-dimensional (3D) printing of magnetic composites. To improve the composite’s ferromagnetic behavior, the particles were aligned along the stacking direction of the layers during the 3D FDM process by printing directly onto a permanent magnet placed on the build plate. Composites containing up to 50% by weight of recycled NdFeB powder were successfully processed using FDM technology, exhibiting increased stiffness, with the storage modulus rising from 123 to 178 MPa at 20 °C, while magnetic field-assisted printing increased the remanence from 11 to 28 emu/g and improved the reduced remanence from 0.21 to 0.49, corresponding to an estimated fourfold improvement in the magnetic energy product.

## 1. Introduction

Countless electrical and electronic tools are made up of components that use rare earth-based magnetic materials containing Neodymium (Nd), Samarium (Sm), and Dysprosium (Dy). The most used alloy to produce permanent hard magnets is composed of Neodymium, Iron, and Boron intermetallic compounds (NdFeB). These permanent magnets play an essential role in the technological transition to a greener economy based on the production, transfer, and use of energy in the form of electricity, and therefore, their demand is constantly growing [1]. The market value of permanent magnets was valued at USD 20.58 billion in 2022, with a compound annual growth rate (CAGR) close to 8.6% from 2023 to 2030. Demand is growing for electronic products such as smartphones, laptops, and air conditioner motors [2].

The growing demand for these materials in various industrial sectors, especially in the automotive (electric cars), wind energy (such as wind turbines [3]), electronics, and automation fields, leads to considerable market instability and sudden changes in raw material prices [4]. These materials are characterized by a high economic impact but also by a high procurement risk due to the distribution and availability of raw materials in the producing countries and also concerning their geographical position [5]. Secondly, these materials could also be considered co-responsible for the tendency of many European companies to relocate their activities to the places of extraction and production of rare earth-based alloys [6]. It is also necessary to consider the influence of governmental stability and economic agreements with the extracting countries on this type of market. Furthermore, the environmental problems of the extraction of these elements from mines, which involve, among other consequences, an increase in CO_2_ emissions, should be mentioned [7]. Considering the current geopolitical situation, characterized by a tendency towards protectionism, and the decline in raw materials as well as finished products, the possible availability of valuable secondary raw materials could overall constitute an advantage for the member countries of the European Union far beyond the simple aspect of environmental sustainability. The recovery of materials from waste electrical and electronic equipment (WEEE) is of crucial importance due to the global crisis in rare earth supply [8].

The high use of permanent magnets in data storage, such as Hard Disks (HDs), is starting to slow down due to the development of increasingly cheaper solid-state data storage systems (SSD), which, in the medium-long term, will replace a large part of the production of HD permanent magnets.

Therefore, reuse by demagnetization and re-magnetization in new HDs is not a sustainable strategy for the future. It therefore becomes strategic to sustainably recover rare-earth elements from this important source of secondary raw materials for other and more innovative applications, which are currently expanding, such as magnetic sensors [9], wearable electronic devices [10], and soft robotics [11].

At present, however, HD magnets, once recovered, cannot immediately be reused in applications other than HDs, as it is not possible to simply vary their shape. The need to find an environmentally friendly and cost-effective method to reuse rare earth magnets as secondary raw materials has driven the present work, as well as several publications that appeared in recent years [12,13,14,15].

The objective of this research is to develop a novel series of bonded magnets that utilize secondary raw materials more efficiently and cost-effectively, thereby producing a new highly flexible material (magnetic composites of complex shapes).

Several techniques are proposed to recover the magnetic material and reintroduce it into the new production chain. In general, once the device containing the magnet has been identified, there are three possibilities for reusing it:

1. Demagnetize the part, transfer it to a new product of the same or another application, and re-magnetize it. This approach can be identified with the generic term “reusing”.

2. Processing the material to separate the raw materials and reusing them for new applications.

This option represents a type of indirect recycling, indicated by the term “downgrading”, which involves a greater expenditure of energy, because it is necessary to separate the components of the alloy (e.g., chemical dissolution).

3. Recycling the magnet by grinding it to get magnetic alloy powder again.

Inside the HD, the magnet is only a small volumetric fraction compared to the totality of the component [16]. For this reason, the most efficient recycling method passes through the removal of the magnet from the frame, thus avoiding the drawbacks caused by grinding the entire HD to later fractionate the materials.

All the activity within this work is configured as an act of true recycling of the material intended as a NdFeB alloy, being difficult to implement both the reuse of components having particular shapes (type 1 recovery), as well as the separation of the individual elements (type 2 recovery), in conditions of economic and environmental sustainability. The third option seems to be the most promising one since it presents reduced environmental impact and costs (compared to the other techniques). The approach applied in the present work proposes a substantial advancement of the recycling methodology (type 3 recovery), selected as the most sustainable of the three, by proposing mechanical grinding in a protected and inert environment to avoid any oxidation, which would degrade the magnetic properties. This procedure envisages reducing the material to be recycled to a powder in a controlled manner and subsequently converting it into the desired shapes as a 3D magnetic composite to obtain additional magnet specimens for use in new products, thereby overcoming the constraints of reusing (type 1 recovery).

The magnets present inside the HDs are currently almost exclusively sintered materials, i.e., compacted and subsequently treated at high temperatures in a controlled atmosphere to densify and acquire adequate mechanical and magnetic properties. In this treatment, the material is not melted but heated sufficiently to enable atomic diffusion to eliminate voids among powder grains and to produce substantial microstructural changes in the alloy during subsequent cooling. At the same time, the best magnetic and mechanical properties are obtained.

Embedding magnetic particles into a polymer matrix leads to magnetically active composites with reduced specific weight, since polymers are lighter than the conventional metal alloys used to produce permanent magnets. Also, the cost of material production, processing, and handling is substantially reduced [17]. The production and use of magnetic composites for general purpose applications are already robust, and studies involving different types of magnetic materials (NdFeB, SmFeN, Ferrites, etc.) with different polymer matrixes, either thermoplastic (PA, PU, and PEEK) or thermoset, are available [18,19]. Moreover, the route of 3D printing promotes a further step forward, since it allows for more complex shapes and customized products for each application [20]. Furthermore, 3D printing is currently one of the most promising technologies for the recycling and upcycling of plastic materials: recent research highlights how household, industrial, and composite waste can be reused to produce functional components, contributing to the transition toward a circular economy model [21]. Experimental studies show that even reinforced composite materials can be valorized through 3D printing: for example, glass fiber-reinforced polypropylene waste can be transformed into 3D printing filaments with competitive mechanical properties [22]. This approach opens interesting prospects for the reuse of waste from the automotive, marine, and energy sectors, reducing both the environmental impact and consumption of virgin raw materials.

The use of magnetic composites becomes crucial for the recycling method proposed, as they require the particle size of the NdFeB powders to be higher than the magnetic powders used for sintering. The consequent reduction in the specific surface area (coarser powders) reduces the specific reactivity of the powders to oxygen (with a consequent reduction in the fire/explosion risk of the powders during the process). Given this reduced specific reactivity, it becomes technologically sustainable to proceed with the grinding and premixing of the magnetic composites in an inert atmosphere, keeping the percentage of oxidized material at low levels to avoid the use of reducing atmospheres, as this occurs instead during the sintering of the magnets (thus avoiding the use of H_2_ in the process) [23,24]. In this way, it is possible to develop a cheaper and more attractive recycling route for rare earth-based magnets.

Innovative technologies are available to produce magnets from powders that do not involve sintering. They are suitable to produce lesser-performing components from a magnetic standpoint but that are equally interesting from an industrial and technological point of view, such as, for example, magnetic composites composed of a thermoplastic or thermosetting matrix and NdFeB powder mixed. The latter approach is currently booming and derives its considerable industrialization potential from traditional processing technologies for plastic materials, such as injection molding or thermoforming. The possibility of implementing this technology in the currently arising world of 3D printing (additive manufacturing) is also not negligible.

The main advantage of 3D printing lies in the possibility of creating complex shapes and optimizing magnetic properties according to experimental needs compared to traditional ceramic magnets. The 3D-printed magnetic composites allow the creation of customized components from a functional and structural point of view: in fact, they allow the magnetic characteristics to be varied over a wide range, thanks to the amount of polymeric binder and any other additives that bond the magnetic metal particles. They are easier to work with than sintered materials. Furthermore, the possibility of choosing from a wide range of polymeric matrices allows for the creation of magnetic structures with different mechanical characteristics, from rigid to flexible, depending on the type of application. Magnetic composites also open the way to the further technological possibilities of composite materials: an example is hybrid magnets, partly made up of permanent magnetic materials and partly of soft magnetic materials. Given the importance of 3D printing techniques in the generation of components with a complex shape that are capable of maximizing the magnetic characteristics of the material in terms of design optimization, this work proposes the creation of flexible magnetic composites employing FDM 3D printing. The NdFeB-based magnetic powders, obtained from the recycling of discarded hard drives, were ground and sieved until the particle size was reduced to less than 50 μm. Subsequently, these powders were incorporated up to 50 wt% into a highly flexible thermoplastic matrix, polybutylene adipate-co-terephthalate (PBAT), via a twin-screw extrusion process. The composite material thus produced was pelletized and extruded into calibrated filaments, which were subsequently used in FDM 3D printing of the magnetic composites. The resulting samples were then analyzed from microstructural, mechanical, and magnetic perspectives.

The results of the magnetic characterization highlight that applying a magnetic field during the printing process, oriented along the direction of material deposition, improves the magnetic properties of the resulting composites.

Furthermore, the presence of NdFeB powder contributes to improved dynamic mechanical properties, potentially broadening the material’s range of applications.

## 2. Materials and Methods

### 2.1. Sample Preparation

**Figure 1:**
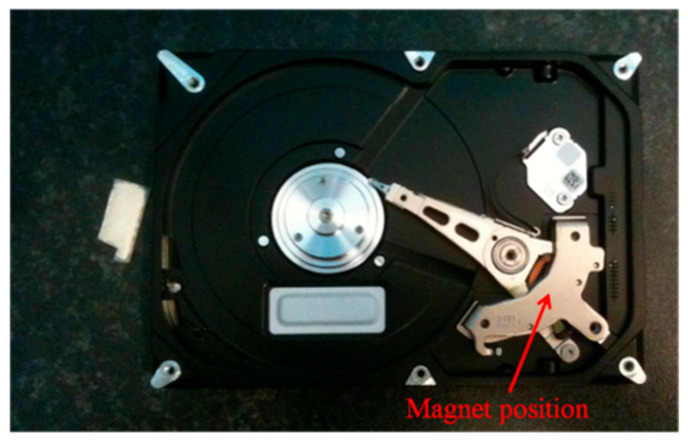
Example of a support surrounding a pair of NdFeB magnets.

**Figure 2:**
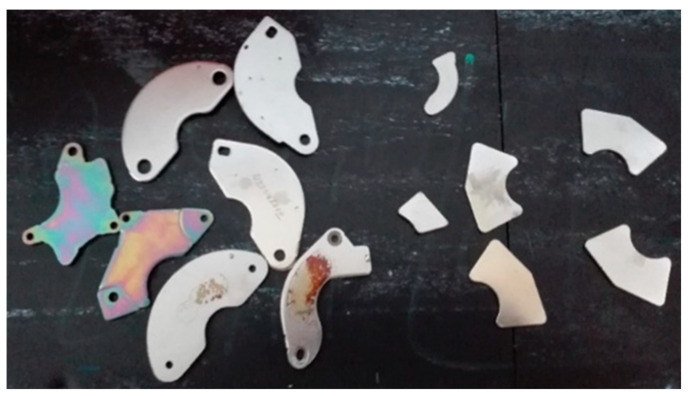
NdFeB magnets (**right**) of different sizes and their magnetic shields (**left**).

The NdFeB-based permanent magnets used in this work derive from HDs collected and supplied by RISTA s.r.l. (Turin, Italy), an Italian Company working in the field of waste recovery and environmental sustainability. OSAI Automation System (Parella, Italy), a leading Italian company in the field of automation and laser for industrial processes, developed an automatic precision system to disassemble the magnets from their supports. An example of magnets mounted and surrounded by magnetic support is shown in Figure 1. The magnets differ from each other in dimension and shape (Figure 2), as well as in chemical composition and magnetic properties. All magnets were ground together, obtaining a powder mixture with average properties. Due to the requirements of mixing with PBAT and subsequent extrusion from the FDM printer, only the finest fraction below 50 μm was used here.

Before the grinding process, the magnets were fully demagnetized at 350 °C for 30 min in a vacuum to avoid oxidation. The thin resin layer, which glued the samples to the supports, degraded in the same phase. After heat treatment, the magnets were easily separated from the supports.

The crushing and grinding phases were performed using a homemade vacuum impact mill, equipped with a jacket for a 10^−2^ mbar vacuum. The mill is composed of two chambers. The first chamber hosts a brushless motor without pole pieces (slot-less type), with a maximum power of 104 W at a speed of 8000 rpm, mechanically sealed off from the second chamber, which contains the magnets to be ground, and the obtained powder. Sealing gaskets are used to keep the vacuum. The process consists of crushing and grinding for 1 min to prevent the motor from overheating, then repeating several times to obtain the required quantity. All produced powders were stored under vacuum to prevent oxidation.

**Figure 3:**
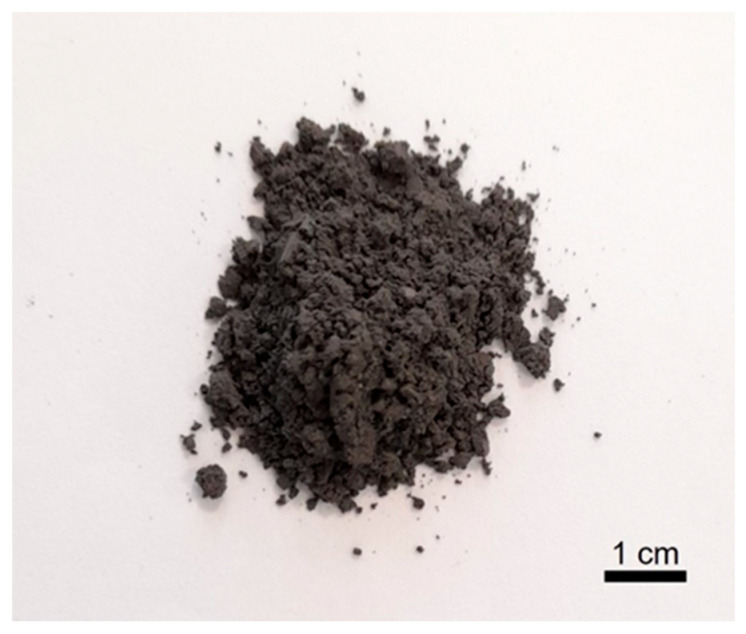
Recycled and sieved NdFeB powder.

The obtained powder was then sieved to isolate the fraction below 50 μm at room conditions (Figure 3) and subsequently dispersed in the thermoplastic matrix.

Polybutylene adipate-co-terephthalate (PBAT) was purchased in pellet form from MAgMa Spa (Chieti, Italy) and utilized in the present work as the thermoplastic matrix of the magnetic composites.

The PBAT granules were ground using an analytical mill (IKA A11 basic, IKA-Werke GmbH & Co. KG, Staufen, Germany) in cryogenic conditions for 1–2 min.

Ground polymer and magnetic powder were pre-mixed in the analytical mill under room conditions. In addition to the pure polymer, two different formulations were prepared, respectively containing 30 and 50% by weight of magnetic powder.

The pre-mixed compositions were processed using a co-rotating twin-screw extruder (RES-2P/12 Explorer Extruder, Zamak Mercator, Skawina, Poland), obtaining a composite filament as a semi-finished product, which was subsequently pelletized into granules. The temperature profile in the heating zones of the screws is T_head_ = 115 °C, _Tzone 6_ = 115 °C, T_zone 5_ = 125 °C, T_zone 4_ = 130 °C, T_zone 3_ = 125 °C, T_zone 2_ = 118 °C, T_zone 1_ = 110 °C, and T_hopper_ = 40 °C, with the screw speed equal to 20 rpm and the maximum load on the screws not exceeding 80%. These conditions were kept constant for all of the compositions.

**Figure 4:**
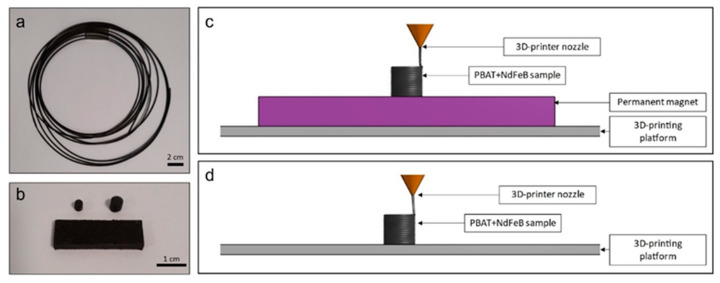
The magnetic filament loaded with 50% NdFeB, as a representative sample (**a**); some of the 3D-printed samples (**b**); 3D printer configurations with (**c**) and without (**d**) the permanent magnet on the printing platform.

The granules obtained after twin-screw extrusion were used to get the constant-diameter filament suitable for 3D printing. For this purpose, a benchtop extrusion system (Felfil Srl., Turin, Italy), composed of a single screw extruder, equipped with a cooling fan array, and a spooler with optical control, was utilized. For filament production, the extruder temperature of 145 °C, the screw speed of 3–5 rpm, and the winding speed of about 1 m/min were used. The obtained 50% loaded filament, as a representative sample, is shown in Figure 4a.

The 3D-printed specimens, designed with SolidWorks software (2024), have a parallelepiped shape of different sizes: 30 × 10 × 2 mm^3^ for dynamic mechanical measurements, and ca. 6 × 6 × 2.5 mm^3^ for magnetic characterizations.

The objects were obtained using a Prusa i3 MK3S+ FDM 3D printer (Prusa Research, Czech Republic) equipped with a 0.4 mm nozzle, setting the printing parameters in the PrusaSlicer software (PrusaSlicer 2.7.4). Specifically, layer height was set at 0.2 mm and strand width was set at 0.35 mm, infill density and geometry were set to 100% and linear, respectively, alternating the infill direction by 90° between adjacent layers, the printing speed was set to 50 mm/min, and finally, extrusion flow was set to 125%. The main 3D-printing parameters were set as follows: nozzle temperature was equal to 180 °C, bed temperature was not heated, and printing speed was fixed in the range of 20–50 mm/s. The printing conditions for the composites were optimized based on laboratory tests conducted on pure PBAT.

FDM 3D printing was also carried out in the presence of a permanent magnet to increase the magnetic strength of the magnetic composites. In this way, the molten material coming out from the nozzle of the 3D printer was surrounded by a magnetic field that allowed for the anisotropic orientation of the magnetic particles embedded in the material, as displayed in Figure 4c,d. The permanent magnet used in 3D printing is a sintered NdFeB magnet with the following characteristics: remanence (Br) = 1.17 T; intrinsic coercivity (HcJ) = 960 kA/m; coercivity (HcB) = 840 kA/m; and maximum energy product (BHmax) = 250 kJ/m^3^.

The produced samples, at different NdFeB powder contents with and without magnetic support, are summarized in Table 1 and are shown in Figure 4b.

### 2.2. Sample Characterization

NdFeB particle size distribution was measured using a Mastersizer 3000 laser granulometer (Malvern Instruments Ltd., Malvern, UK) according to ISO 13320 [25].

Composite infrared (IR) spectra were acquired on a Spectrum Two FT-IR spectrometer (Perkin Elmer, High Wycombe, UK) in attenuated total reflectance (ATR) mode.

Scanning electron microscopy (SEM) characterizations were performed using a field emission SEM (FESEM, Nova NanoSEM 450, FEI company, Morristown, NJ, USA). Backscattered electron images were collected to highlight the dispersion and distribution of NdFeB particles in the polymer matrix. The accelerating voltage of 12 kV, spot size of 3 a.u., and a working distance of about 6 mm were utilized in the acquisition of all images.

The elemental composition of the magnetic composite was obtained at the same time as the SEM analyses using the energy-dispersive X-ray spectroscopy system (X-EDS) QUANTAX-200 (Bruker, Ettlingen, Germany). X-ray diffraction (XRD) and X-ray fluorescence (XRF) analysis on powder and composite samples were carried out using a Bruker New D8 ADVANCE ECO diffractometer (Bruker, Ettlingen, Germany) equipped with a Cu Kα (1.5406 Å) radiation source and a Shimadzu EDX-7000 (Shimadzu, Kyoto, Japan), respectively.

Dynamic mechanical properties were determined using a TA Q800 dynamic mechanical analyzer (DMA) (TA Instruments, New Castle, DE, USA) on the 3D-printed specimens of rectangular geometry. Samples were tested in flexure using the single cantilever clamp configuration. The measurement conditions were the following: the frequency was 1 Hz, the amplitude was 10 μm, and the temperature range was chosen between −55 °C to 80 °C with a 3 °C/min heating rate.

Magnetic characterization was performed at room temperature using a Superconducting Quantum Interference Device (SQUID) by Quantum Design Ltd. (San Diego, CA, USA), operating in the 2–400 K temperature range and up to 5 T applied magnetic field. SQUID magnetometers allow very precise measurements of the magnetic moment, with a sensitivity in the order of 10^−7^ emu. Conversely, the maximum moment value they can measure is limited to a few emus. For this reason, this instrument is adequate for small-sized samples only. To avoid signal saturation, the size of the measured sample was adapted by cutting a larger pristine cube of prepared material down to a small parallelepiped of about 6 × 6 × 2.5 mm^3^. All magnetic measurements were carried out by aligning the magnetic field of the instrument to the smaller side of the paralepidid, also corresponding to the magnetic orientation of the NdFeB powder induced by the permanent magnet during the 3D printing fabrication of the oriented samples. A pellet of a few milligrams of pristine powder was also measured as a reference. The magnetization value was normalized to the mass of the sample, measured by high-precision balance (resolution 0.01 mg).

## 3. Results and Discussion

**Figure 5:**
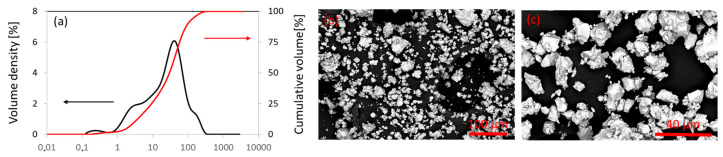
Distributive and cumulative particle size distribution curves (**a**) and SEM image of NdFeB powder (**b**,**c**) at different magnifications.

The particle size curves, distributive and cumulative, of NdFeB powders are shown in Figure 5a. The characteristic distribution parameters are D10 = 2.7 ± 0.5 µm, D50 = 26 ± 2 µm, and D90 = 88 ± 1 µm, with the volume weighted mean, Dmean, equal to 38.5 ± 0.9 µm. Closing the curve below 100 µm, the difference in values compared to the sieving process could be due to an aspect ratio of the powder particles greater than 1 or to magnetic interactions between the particles with the consequent formation of aggregates, detected by the granulometer as a single structure. The validity of such assumptions is supported by SEM images, which show an irregular morphology, with the presence of more complex NdFeB structures consisting of magnetic grain aggregates (Figure 5b).

**Figure 6:**
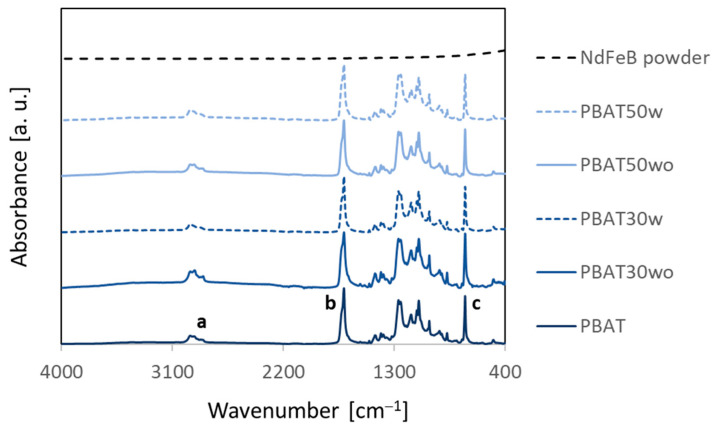
IR spectra of pristine PBAT, different magnetic composites, and NdFeB powder. Labels a, b and c indicate the main absorption bands of the polymer.

Figure 6 shows the IR spectra of magnetic composites at different NdFeB powder concentrations, indicating no structural variation in the polymer matrix due to manufacturing processes or interactions with magnetic powder. Pristine PBAT and pure NdFeB powder are also shown as references.

IR spectra confirm the PBAT structural characteristics already seen previously [26,27]: the symmetric and asymmetric stretching of the methylene group in adipate and butanediol units (label “a” in Figure 6—1950–1850 cm^−1^), the stretching of the carbonyl group in the adipate and terephthalate units (label “b” in Figure 6—1705 and the shoulder at 1730 cm^−1^), and the out-of-plane bending in the aromatic ring of terephthalate units (label “c” in Figure 6—around 725 cm^−1^) can be identified as representative peaks, while NdFeB powder does not show characteristic absorptions in the analyzed wavenumber range.

**Figure 7:**
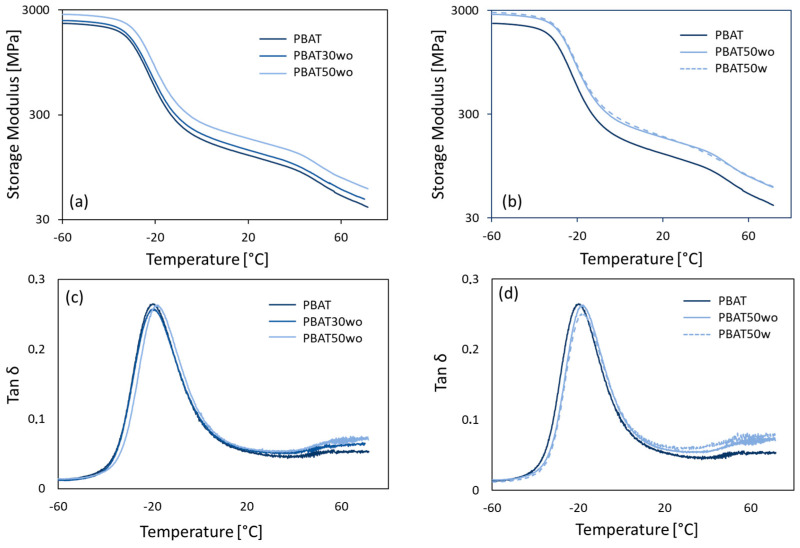
DMA and Tan δ curves of magnetic composites at different NdFeB powder contents printed without (**a**,**c**) and with (**b**,**d**) the magnetic support.

The thermomechanical properties of the magnetic composites were analyzed by DMA between −60 and 80 °C, in a thermal range covering the glassy and rubbery zones of the polymer matrix. The conservative moduli, E’, of the filled materials PBAT30wo and PBAT50wo, increase as the NdFeB powder content increases throughout the considered thermal range (Figure 7a). The increase in E’ as the NdFeB content increases confirms the reinforcing effect of the rigid magnetic particles within the PBAT matrix. In particular, the modulus at 20 °C increased from 123 MPa for pure PBAT to 178–180 MPa for composites containing 50 wt% NdFeB, corresponding to an improvement of approximately 45%. A similar stiffening effect has been widely reported in the literature for polymer-bonded magnetic composites and is generally attributed to the reduced mobility of the polymer chains caused by the presence of rigid inorganic fillers. Comparable trends were observed by Slapnik et al. [18] for 3D-printed NdFeB-filled composites based on PA12 and TPU matrices, where the elastic modulus increased proportionally to the magnetic filler loading.

The magnetic support during printing does not seem to influence the mechanical performance of the magnetic composites: in Figure 7b, the PBAT50w and PBAT50wo formulations are represented as representative samples. The negligible differences between PBAT50wo and PBAT50w indicate that the magnetic field-assisted printing process does not significantly affect the material’s thermomechanical response. This suggests that the particle orientation induced during FDM primarily influences magnetic anisotropy rather than the viscoelastic properties of the polymer matrix. Similar conclusions have been reported in recent studies on magnetic composites produced using additive techniques, in which magnetic alignment improved remanence and magnetic performance [20].

Also, the glass transition temperature, Tg (calculated as the peak of the Tan δ curve), does not seem to vary appreciably as a function of both the NdFeB powder content and the printing configuration, indicating a poor interaction between the PBAT and the NdFeB particles: the small variations observed are not significant, falling within the range of experimental standard deviations (Table 2). In these conditions, the polymeric matrix still maintains its thermal stability; indeed, the conservative modulus, which increased following the addition of the magnetic charge, allows for an increase, even if modest, of the thermal range of material applicability, considering the room temperature as a reference. Table 2 shows the E’ values at 20 and 40 °C, which define a probable thermal range of the material used.

Similar behavior has been observed in other thermoplastic composites reinforced with metal or ceramic particles, where the lack of significant compatibility between the filler and the matrix resulted in negligible changes in Tg despite substantial increases in stiffness. Maintaining the Tg is technologically advantageous, because it allows for an increase in mechanical stiffness without compromising the flexibility typical of PBAT.

The observed behavior is like that found for other polymer-based composite systems loaded with rigid fillers, with limited interactions at the filler–matrix interface [28].

**Figure 8:**
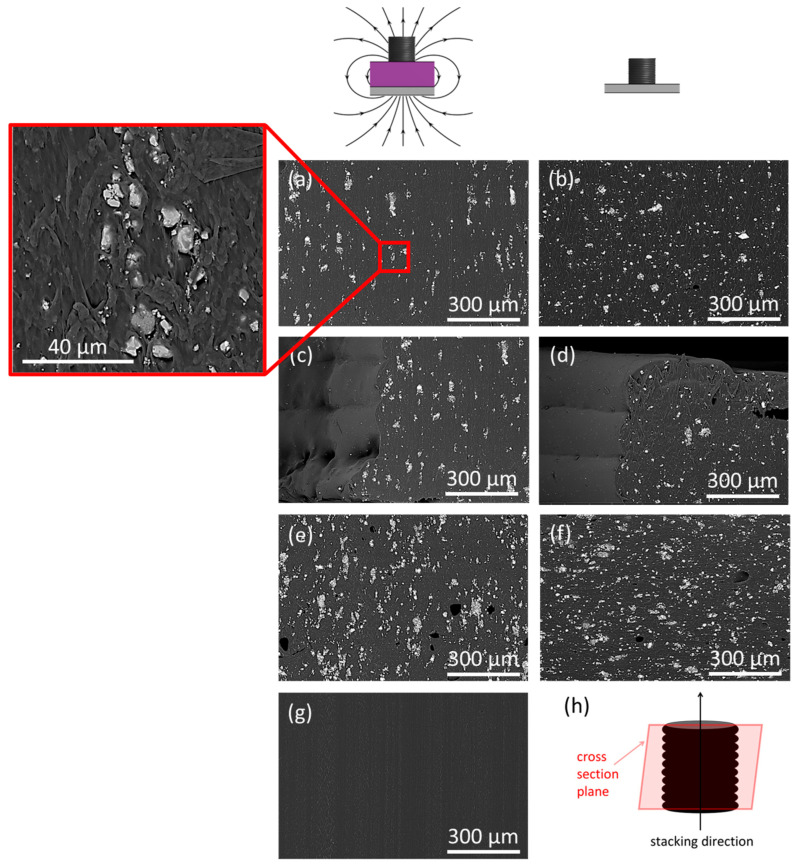
SEM images of PBAT30w (**a**)—in the inset, NdFeB particles in the PBAT30w sample at higher magnifications, PBAT30wo (**b**), side view of PBAT30w (**c**), side view of PBAT30wo (**d**)—as representative samples, PBAT50w (**e**), PBAT50wo (**f**), and not-loaded PBAT (**g**); graphic representation of analyzed surfaces (**h**). On the top, the two printing configurations are schematized, with the magnetic field lines generated by the permanent magnet.

The SEM images (Figure 8) show the distribution of NdFeB particles (white spots) in the PBAT matrix and refer to the sample section parallel to the stacking direction of the layers (Figure 8h). In magnetic-aided printed composites, a preferential particle orientation can be observed, with the particles oriented along the direction of the external magnetic field lines (Figure 8a,e).

The orientation of the NdFeB particles is essentially parallel to the stacking direction of the different layers, as can be seen in the side-view images of the 30% PBAT-filled samples (as representative samples), printed with and without permanent magnetic support (Figure 8c,d). No influence of the external magnetic field on the interlayer bonding can be noted between two adjacent layers, which are nevertheless well linked to each other in both printing configurations.

Conversely, in the samples printed with the standard configuration, the NdFeB particle distribution in the polymeric matrix is random (Figure 8b,f). For comparison, the microstructure of the PBAT matrix is also reported (Figure 8g).

**Figure 9:**
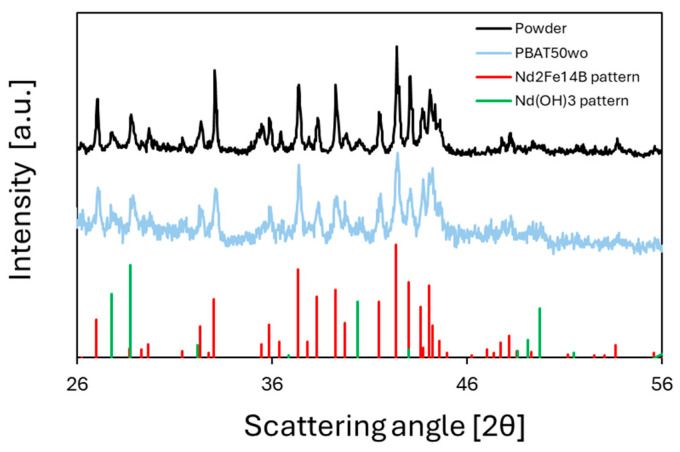
Comparison of the XRD spectrum of the composite (blue line) with that of the recycled powder sieved below 50 mm (black line). NdFeB (red bars) and Nd(OH)_3_ (green bars).

An analysis using the XRD technique confirmed that the recycled powder did not lose the pristine crystallographic properties after the sieving process and mixing with PBAT. In Figure 9, the XRD patterns of the sieved powder and PBAT50wo, taken as a representative example, are shown together with the NdFeB reference. A small amount of Nd(OH)_3_, estimated at 5–7% by Rietveld analysis, can also be observed with XRF analysis (Table 3), which showed that the Fe to Nd molar ratio is preserved in the composite and is close, within the accuracy of the measurement (about 1%), to that expected for the NdFeB alloy. Elemental analysis also shows that the samples contain Co and rare earth elements in residual percentages (<5%). The presence of Nd(OH)3 is likely due to oxidation on the active surface of NdFeB, caused by residual water and oxygen during ball milling or subsequent processes.

To check the magnetic properties of the composites and to compare them with the pristine powder of recycled NdFeB, the most loaded samples (PBAT50w/wo) were measured with a SQUID magnetometer.

**Figure 10:**
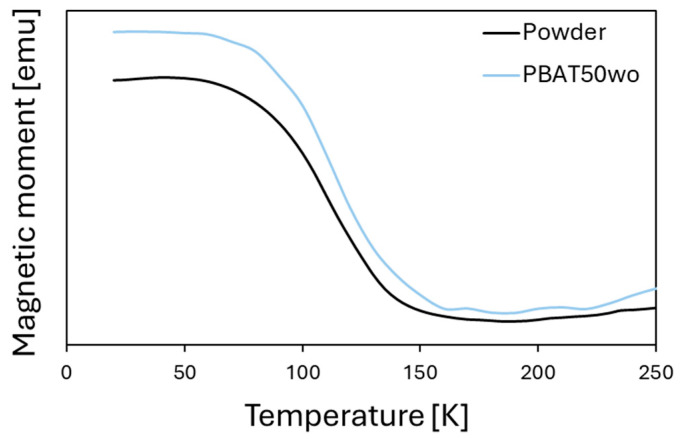
Magnetization of composite PBAT50wo, as representative sample, (blue line) and powder (black line) as a function of the temperature measured with a constant magnetic field of 1 KOe.

The measurement of the magnetization as a function of the temperature with a 1 kOe field (Figure 10) evidenced a sharp decrease in the range 100–150 K for both the NdFeB powder and the PBAT composites: this behavior can be associated with the Spin Reorientation Transition typical of this material [29].

**Figure 11:**
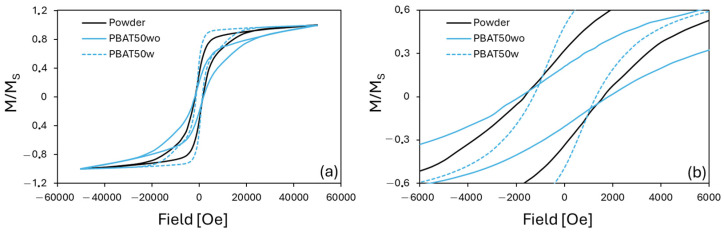
Hysteresis loops for PBAT50w/wo as representative samples (**a**) compared to that of the pristine powder, normalized to Ms, as reported in Table 4. Details of the curves in the low field range (**b**).

In Table 4, the main magnetic parameters such as saturation magnetization (Ms), magnetic remanence (Mr), and the coercivity (Hc) of PBAT50w/wo samples were reported and compared with those of the powder. The saturation magnetization, Ms, associated with the maximum value of M measured at 5 T, is a little lower than expected considering the nominal powder percentage in the PBAT (Ms_PBAT50% = 40% Ms_powder), suggesting that part of the powder was lost in the embedding procedure or was not well mixed in the PBAT. The complete hysteresis loops at 300 K of PBAT50w/wo are shown in Figure 11, together with the hysteresis of the powder. For a better comparison of the curve shape, M is divided for the corresponding Ms values, as reported in Table 4. The shape of the curves shows a clear difference depending on the initial orientation of the magnetic powders. In the oriented sample (w), since the easy axis is parallel to the applied field, the magnetization easily reaches saturation (the almost-flat behaviors from 1 T up) and hardly loses the acquired magnetization when the applied field is removed (high remanence) [30]. The remnant magnetization suddenly drops down only when the direction of the field is inverted and the anisotropy is overcome. This confers a “squared” shape to the hysteresis loop, as observed. Conversely, in the not-oriented printing condition (wo), the random orientation of the easy axis of the particles produces a gradual increase in the magnetization with the increase in the applied field because of the anisotropy energy barrier, which is due to the portion of the easy axis oriented perpendicularly (or, in general, not parallel) to the external field direction. For this reason, the not-oriented samples do not completely reach magnetic saturation in the range of the applied fields, and the hysteresis loop shape is more “elongated” compared with the previous case [31]. The different shape of the hysteresis loops implies a higher coercivity and lower remanence in the randomly oriented sample [32].

The powder hysteresis curve shows intermediate behavior, probably because of a partial orientation of the grains, which are not completely blocked in a polymetric matrix with isotropic orientation like the PBAT50wo sample, but can partially align under the effect of internal interactions and pressure during sample preparation and under the magnetic field action during measurement [33].

The magnetic measurements indicate that the NdFeB powder retains most of its properties once embedded in the polymeric matrix and that the orientation of its grains is important to tune the shape of the hysteresis. It is well known, indeed, that the squareness of the hysteresis loop is one of the most important magnetic features to maximize the energy product [32,34].

For such small samples, a reliable direct assessment of the energy product (BHmax) was not possible, primarily because the real density and the demagnetization factor, a key parameter for evaluating the internal field and therefore BHmax, cannot be accurately estimated. However, a rough estimate of the ratio between BHmax for oriented and non-oriented samples, considering that only the Mr and Hc values differ between the two samples, showed that BHmax (w) is about four times BHmax (wo) [35].

Besides the orientation of the magnetic grain, another important parameter is the weight ratio of the NdFeB powder, which determines the value of its magnetization, and, consequently, the BHmax. From this point of view, the magnetic composites are expected to have lower magnetic properties than the magnetic materials obtained by conventional methods (e.g., sintering) or bound magnets with a high magnetic load (90–95%) but greater ability to be molded into complex shapes by FDM 3D printing [17,36].

The preparation procedure described here demonstrated the possibility of producing a composite magnetic material with properties like those of the original powder, which can be molded into the desired shapes using a 3D printer. It was also shown that grain orientation, easily achieved during the 3D process, can significantly improve the BHmax of the composite.

Since the magnetic performance of the final composite strongly depends on the powder used in the composite, further efforts must be made to improve the magnetic properties of the powder, which, in this case, are rather poor. The first improvement is to find a compromise between the maximum grain size and the extrusion needs. Although the recycled material was found to have the compositional characteristics of NdFeB, the hysteresis curves showed a significant difference between the sieved and unsieved powder, mainly in the coercivity [37]. The main features of the NdFeB bulk, like coercivity and remanence, are indeed better retained in larger grains, which are less exposed to oxidation due to their lower surface/volume ratio. However, even the magnetic properties of the unsieved recycled powder are lower than those of the NdFeB bulk. A more significant improvement will be thus achieved by optimizing the grinding method.

## 4. Conclusions

New technologies are available for producing magnetic materials from recycled powders without sintering. They are suitable for producing less magnetically high-performance components, but they are still interesting from an industrial and technological perspective. For example, magnetic composites made from a thermoplastic matrix and NdFeB powder enable the production of on-demand components with modulated magnetic properties depending on the composition. The implementation of this technology in the rapidly growing field of additive manufacturing should not be ignored. This technology can produce complex-shaped components that optimize the magnetic characteristics of the material for design purposes. The main findings of the study can be summarized as follows:

- PBAT-based magnetic composites were obtained by FDM 3D printing;
- NdFeB powder content has been increased up to 50 wt% in the composite, making these materials interesting for applications in sensing and magnetic storage;
- To increase the magnetic properties, a permanent magnet has been used as a printing base during the manufacturing process;
- The results of magnetic measurements indicate that higher remanence values can be achieved by producing the magnetic composites with a magnetic field applied during the printing process and aligned with the printing direction;
- This configuration is of great interest and can still be applied to future research efforts. Furthermore, it is possible to reduce the polymer content to increase the NdFeB powder load, thereby improving the magnetic properties and expanding the material’s potential applications.

In light of the growing interest in polymer composites and additive manufacturing technologies, this study provides a valuable foundation for future experimentation and the development of commercially relevant multifunctional components and polymer-based magnetic composites.

## Figures and Tables

**Table 1:** Sample names and compositions.

Sample Name	NdFeB Powder Content [wt%]	3D Printer Configuration
PBAT	/	without magnet
PBAT30wo	30	without magnet
PBAT30w	30	with magnet
PBAT50wo	50	without magnet
PBAT50w	50	with magnet

**Table 2:** Conservative moduli, E’, of magnetic composites at lower (−50 °C) and higher (20 and 40 °C) temperatures than the Tg, also tabulated.

Sample	E’ @ −50 °C [MPa]	E’ @ 20 °C [MPa]	E’ @ 40 °C [MPa]	T_g_ [°C]
PBAT	2179 ± 20	123 ± 5	90 ± 3	−19.2 ± 1.9
PBAT30wo	2317 ± 21	137 ± 9	100 ± 9	−19.6 ± 1.2
PBAT50wo	2682 ± 5	178 ± 3	131 ± 3	−18.4 ± 0.4
PBAT50w	2760 ± 15	180 ± 3	128 ± 3	−18.5 ± 0.4

**Table 3:** Elemental composition (% *w*/*w*) resulting from XRF analysis performed in air. Boron, being a light element, was not detected, because its low-energy fluorescence X-rays are easily absorbed by the air or the sample itself. Relative accuracy can be estimated by 1%.

Element	% *w*/*w* Powder	% *w*/*w* PBAT50wo
Fe	66.6	66.7
Nd	29.9	27.7
Tm	0.9	1.6
Co	0.6	1.4
V	0.9	0.9
La	1	1

**Table 4:** Saturation magnetization (Ms), magnetic remanence (Mr), reduced remanence (M_R_), and coercive field (Hc) values obtained from SQUID measurements.

Sample	Ms [emu/g]	Mr [emu/g]	M_R_ [Mr/Ms]	Hc [Oe]
Pristine powder	147	47	0.32	1667
PBAT50w	58	28	0.49	1310
PBAT50wo	52	11	0.21	1852

## Data Availability

The original contributions presented in this study are included in the article. Further inquiries can be directed to the corresponding author.
